# Effects of an 8-week aerobic exercise program on plasma markers for cholesterol absorption and synthesis in older overweight and obese men

**DOI:** 10.1186/s12944-021-01537-2

**Published:** 2021-09-21

**Authors:** S. Mashnafi, J. Plat, R. P. Mensink, P. J. Joris, J. P. D. Kleinloog, S. Baumgartner

**Affiliations:** 1 Department of Nutrition and Movement Sciences, NUTRIM school of Nutrition and Translational Research in Metabolism, Maastricht University, PO Box 616, 6200 MD Maastricht, the Netherlands; 2 Department of Medical Basic Sciences, Faculty of Applied Medical Sciences, AlBaha University, AlBaha, Saudi Arabia

**Keywords:** Aerobic exercise, Cholesterol metabolism, Cholesterol absorption, Cholesterol synthesis, Non-cholesterol sterols, Plant sterols, Cholesterol precursors

## Abstract

**Background:**

Increased physical activity is inversely related to the risk to develop cardiovascular disease (CVD). In a recent systematic review, it was reported that CVD patients had an increased cholesterol absorption and a decreased synthesis as compared with control participants. As increased physical activity levels reduce CVD risk, we hypothesized that exercise training will reduce cholesterol absorption and increase endogenous cholesterol synthesis in older overweight and obese men.

**Methods:**

A randomized, controlled, crossover trial was performed. Seventeen apparently healthy older overweight and obese men were randomized to start with an aerobic exercise or no-exercise control period for 8 weeks, separated by 12 weeks washout. Fasting serum total cholesterol (TC) and non-cholesterol sterol concentrations were measured at baseline, and after 4 and 8 weeks.

**Results:**

The aerobic exercise program did not affect serum TC concentrations. In addition, exercise did not affect TC-standardized serum concentrations of sitosterol and cholestanol that are markers for cholesterol absorption. However, a trend for reduced TC-standardized campesterol concentrations, which is another validated marker for cholesterol absorption, was observed as compared with control. Lathosterol concentrations, reflecting cholesterol synthesis, did not differ between both periods.

**Conclusions:**

Aerobic exercise training for 8 weeks did not lower serum TC concentrations in older overweight and obese men, but a trend towards a decrease in the cholesterol absorption marker campesterol was found. The cholesterol synthesis marker lathosterol did not change.

**Trial registration:**

posted on www.clinicaltrials.gov as NCT03272061 on 7 September 2017.

**Supplementary Information:**

The online version contains supplementary material available at 10.1186/s12944-021-01537-2.

## Introduction

Increased physical activity is inversely related to the risk to develop cardiovascular disease (CVD) [1]. Indeed, aerobic exercise improves several CVD risk markers such as body composition, blood pressure, low-grade systematic inflammation and immune function [2–5]. In addition, some training intervention studies have reported increased serum high-density lipoprotein cholesterol (HDL-C) [6], and decreased serum low-density lipoprotein cholesterol (LDL-C) and triglyceride (TG) concentrations [7]. However, the number of involved study participants in these studies was in general small and a meta-analysis including six randomized controlled trials and data from 192 men and women with intervention periods ranging from 10 to 104 weeks concluded that serum total cholesterol (TC), LDL-C and HDL-C concentrations were not improved after aerobic exercise training [8].

Even without affecting serum TC concentrations, aerobic exercise may still affect processes underlying cholesterol homeostasis. These processes e.g., intestinal cholesterol absorption and endogenous cholesterol synthesis do not only determine serum TC concentrations, but may also be related to certain metabolic diseases. Non-cholesterol sterols have been validated as markers for cholesterol absorption and cholesterol synthesis [9]. Circulating levels of campesterol, sitosterol and cholestanol reflect intestinal cholesterol absorption, while lathosterol and desmosterol levels are markers of endogenous cholesterol synthesis [10]. Using these markers, patients can be classified as cholesterol absorber or synthesizer [11–14]. For instance, chronic kidney disease (CKD) patients on hemodialysis are characterized by higher cholesterol absorption and lower cholesterol synthesis compared to controls [15, 16]. Furthermore, mortality rates in hemodialysis patients with a high cholesterol absorption are higher compared to those of patients with a low cholesterol absorption [16]. Further, in a recent systematic review, it was also reported that CVD patients have an increased cholesterol absorption and decreased cholesterol synthesis as compared with control participants [17]. It can be speculated that aerobic exercise does not lower serum TC concentrations, but may lower the risk to develop CVD by reducing intestinal cholesterol absorption and increasing cholesterol synthesis.

So far, only a few trials have examined the effects of exercise training on cholesterol absorption and synthesis, but results were inconsistent. Using stable isotopes, Varady et al. [18] showed that endurance exercise for 8 weeks did not affect cholesterol absorption and synthesis in hypercholesteremic subjects. However, Wilund et al. [19] observed, after an endurance exercise program for 6 months, an increase in TC-standardized campesterol levels, but no significant change in TC-standardized lathosterol levels. However, a no-exercise control group was lacking. In contrast, Cho and coworkers reported in ten overweight participants that an 8-week intervention of combined resistance and aerobic exercise reduced cholesterol synthesis without changing cholesterol absorption [20]. These discrepant findings might be explained by various factors, such as type of exercise training program, sample size, lack of control group and the methodology to measure cholesterol metabolism markers. Therefore, the purpose of this study was to examine the effect of an 8-week aerobic exercise training period as compared to a control period on markers for cholesterol metabolism in older, overweight and obese sedentary men. The population group of the study was deliberately chosen, as they have an increased risk to develop CVD [21–24]. The hypothesis of the study is that exercise reduces cholesterol absorption and increases cholesterol synthesis, a phenotype that may be associated with a decreased CVD risk [17].

## Methods

### Study participants

Details of this study have been published before [25]. Briefly, nineteen Caucasian men aged between 60 and 70 years were recruited. Subjects were eligible to participate based on the following inclusion criteria: sedentary lifestyle (classified as low physical activity according to the guidelines for International Physical Activity Questionnaire (IPAQ)); body mass index (BMI) between 25 and 35 (kg/m^2^); no smoking; no diabetes; no active CVD; no drug or alcohol abuse; no use of dietary supplements known to interfere with the study outcomes; no use of medication known to affect lipid or glucose metabolism; fasting plasma glucose concentrations < 7.0 mmol/L; fasting serum TC concentrations < 8.0 mmol/L and fasting serum TG concentrations < 4.5 mmol/L. This study was conducted in accordance with the declaration of Helsinki and written informed consent was obtained from all participants before the start of the study.

### Study design

This 8-week, randomized, controlled, cross-over trial was conducted at Maastricht University in Maastricht, the Netherlands from January 2018 to December 2018. Participants were randomized to start with either the exercise period or the control period using a computer-generated, randomization scheme. The protocol was approved by the Medical Ethics Committee of Maastricht University Medical Center (METC173025), and the study was registered on September 7th, 2017 at ClinicalTrials.gov (NCT03272061).

### Exercise protocol

The intervention period consisted of a supervised, progressive, aerobic exercise training 3 times a week for 8 weeks performed on a cycling ergometer. The training intensity was determined for each individual based on their maximal power (Pmax), which was reassessed every two weeks during the exercise period. Each exercise session started with 10 min of warming-up and ended with 10 min of cooling down corresponding to 45% of participant’s Pmax. In-between, the participant cycled for 30 min at 70% Pmax. Participants’ attendance was recorded to assess exercise compliance. At the end of each period, energy and nutrient intakes were calculated for each participant using a validated food frequency questionnaire. During the control period, participants were requested to maintain their habitual activity levels. A wearable accelerometer (activPAL3; PAL Technologies Ltd., Glasgow, United Kingdom) was used to assess physical activity and sedentary behavior for a median duration of 5 days at the end of each period. During this period, no exercise training was performed [25]. The exercise and control periods were separated by 12 weeks washout period, in which participants returned to their habitual activity levels. As previously described [25], peak oxygen consumption (VO_2_ peak) was measured during a maximal exercise test using an incremental step-wise protocol. Measurements were performed every 2 weeks during the exercise period and three times during the control period.

### Serum lipid analysis

Twelve-hour fasting venous blood samples were drawn at baseline, week 4 and at two follow up days in week 8. The first follow up sample was collected 43 h (median: range 19–72 h) and the second follow up sample 117 h (70–118 h) after an exercise training. Blood samples were centrifuged at 1300x g for 15 min at 21 °C to collect serum samples that were stored at − 80 °C until analyzed at the end of the study. Serum TC (CHOD-PAP method; Roche Diagnostic, Mannheim, Germany), HDL-C (CHOD-PAP method; Roche Diagnostic, Mannheim, Germany), and TG concentrations were analyzed with enzymatic methods (GPO-Tinder; Sigma-Aldrich Corp., St. Louis, MO, USA).

### Serum non-cholesterol sterol analysis

Serum non-cholesterol sterol concentrations, campesterol, sitosterol, cholestanol and lathosterol, were measured by gas chromatography (GC) equipped with a flame ionization detector (GC-FID) (Hewlett Packard 6890 plus), using a capillary column (DB-XLB 30 m × 0.25 mm i.d. × 0.25 um; Agilent Technologies, Amstelveen, Netherlands). Extraction of cholesterol and non-cholesterol sterols was performed as described by Mackay et al. [26]. Briefly, 100ul serum sample was saponified with 1 mL of 90% ethanolic sodium hydroxide for 1 h at 60 °C. 5α-cholestane and epicoprostanol were used as internal standards. After two rounds of cyclohexane extraction, samples were derivatized with 30ul of TMS reagent [pyridine, hexamethyldisilazane and trimethylchlorosilane (9:3:1, v/v/v)]. Samples were injected into the GC-FID, and cholesterol and non-cholesterol sterol peaks were integrated (OpenLab CDS ChemStation Edition; Agilent Technologies, Santa Clara, USA), and their concentrations were calculated relative to the internal standard 5α-cholestane concentration. Non-cholesterol sterol concentrations were standardized for cholesterol concentrations, as determined during the same GC run and expressed as umol/mmol cholesterol.

### Statistical analysis

Data are presented as means ± standard deviations (SD), unless indicated otherwise. Normality of distribution was assessed using the Kolmogorov-Smirnov test. Baseline differences in cholesterol and non-cholesterol sterols concentrations between control and intervention periods were analyzed by a paired sample t-test. Changes from baseline over time between the control and intervention periods were analyzed using linear mixed models with treatment and time as within-subject fixed factors and with treatment*time interaction. If the interaction term was not statistically significant, it was omitted from the model. Bonferroni’s correction for multiple comparisons was used, as appropriate. Additional analyses were also performed using linear mixed model to evaluate the difference in changes from baseline in all variables between absorbers and synthesizers subgroups. A *P*-value < 0.05 was considered statistically significant. All data were analyzed using SPSS 24.0 for windows (SPSS Inc., Chicago, IL, USA).

## Results

Of the nineteen men that started the study, two dropped out due to personal reasons. A full consort flow chart is shown in Supplemental Fig. 1. Baseline characteristics of the seventeen men who completed the study are shown in Table 1. The VO_2_ peak values were significantly increased during the exercise period from 2716 ± 454 mL at baseline to 2978 ± 473 mL at week 8, while it was 2713 ± 478 mL at baseline and remained nearly unchanged (2708 ± 459 mL) at the end of control period [25]. These results indicated that cardiorespiratory fitness was increased during the exercise period. BMI and body weight were comparable at the start of the control and intervention periods, and did not change during these periods (data not shown). Physical activity levels, sedentary times and dietary intake between the intervention and control periods were comparable (unpublished data).

**Table 1:** Baseline characteristics (*N* = 17)

Characteristic	Mean ± SD
Age (years)	66.8 ± 1.7
Weight (kg)	94.8 ± 11.7
BMI (kg/m^2^)	30.3 ± 2.8
Glucose (mmol/L)	5.8 ± 0.4
Total cholesterol (mmol/L)	5.3 ± 1.1
Triglycerides (mmol/L)	1.4 ± 0.5

**Fig. 1:**
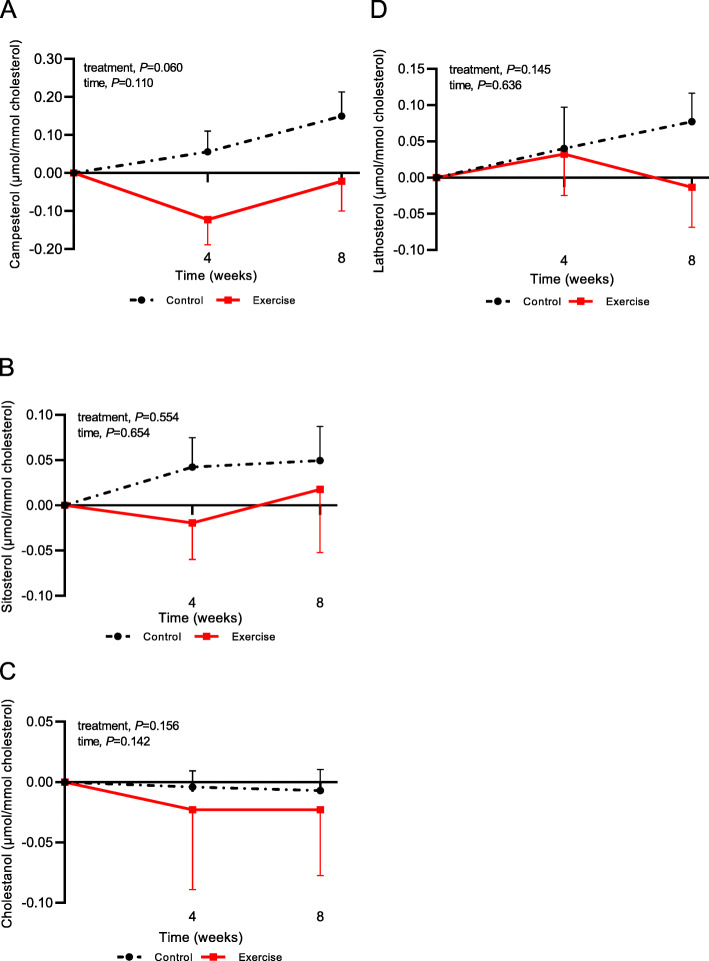
Changes in cholesterol standardized levels of cholesterol absorption markers (**A, B, C**) and cholesterol synthesis marker (**D**) during an 8-week intervention period. Data are presented as mean ± SEM

Serum TC concentrations and TC-standardized serum non-cholesterol sterol concentrations (campesterol, sitosterol, cholestanol and lathosterol) were comparable at the start of the intervention and control periods (Supplemental Table 1). No effects of the aerobic exercise program on serum TC concentrations were observed (Supplemental Table 1). Changes in TC-standardized non-cholesterol levels are shown in Fig. 1. For cholesterol absorption markers, no significant treatment or time effects were observed (Fig. 1, A-C), although TC-standardized campesterol levels tended to be decreased after the exercise period (*P* = 0.060). Levels of the cholesterol synthesis marker lathosterol were comparable between both intervention periods (Fig. 1, D).

**Fig. 2:**
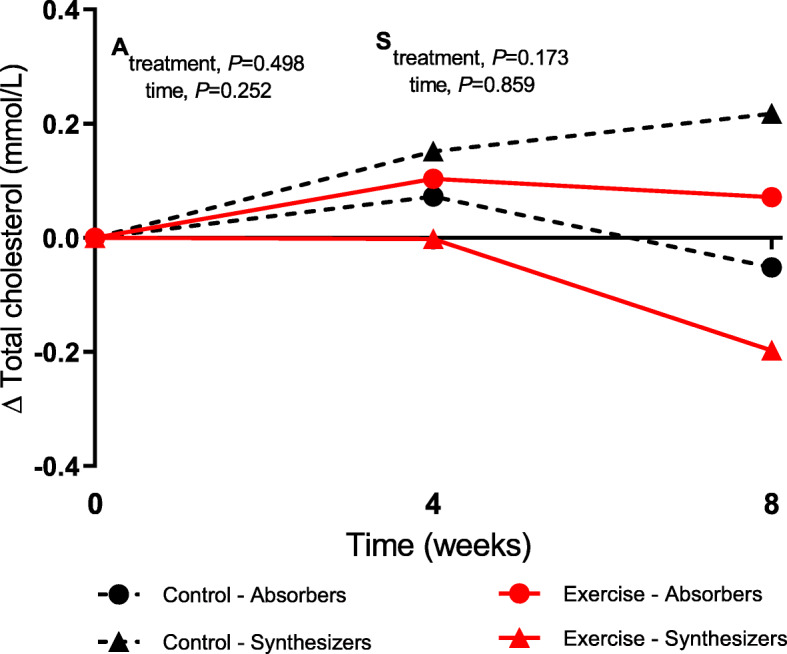
Changes in TC concentrations during intervention periods for cholesterol absorbers (*n* = 9) and cholesterol synthesizers (*n* = 8) subgroups. ^**A**^*P* values for factor effects in cholesterol absorbers. ^**S**^*P* values for factor effects in cholesterol synthesizers

As exploratory analyses, subjects were divided into cholesterol-absorbers and cholesterol-synthesizers based on the median lathosterol-to-campesterol ratio of the group at baseline to investigate differences in responses between these two subgroups after exercise. At baseline, the range in lathosterol-to-campesterol ratio was 0.21–0.69 for cholesterol-absorbers (*n* = 9) and 0.84–1.77 for cholesterol-synthesizers (*n* = 8). Even though changes in serum TC concentrations after aerobic exercise in the cholesterol-synthesizers appeared to be more pronounced than in the cholesterol-absorbers, there were no significant treatment or time effects for TC changes in absorbers or synthesizers (Fig. 2). Also, for changes in TC-standardized campesterol levels, no significant treatment*time effect was observed (*P* = 0.093). However, these levels tended to be lower after exercise in the cholesterol absorbers, but not in cholesterol synthesizers. Changes in other markers showed neither treatment nor time effects in absorbers and synthesizers after exercise (Supplemental Fig. 2).

## Discussion

The present study shows that an 8-week aerobic exercise program did not affect validated plasma markers of cholesterol absorption or synthesis in older overweight and obese men. Based on a recent systematic review [17], it was hypothesized that aerobic exercise training would reduce cholesterol absorption and increase cholesterol synthesis. Although campesterol levels tended to be lower after aerobic exercise, this difference did not reach significance. The two previous studies investigating the effect of exercise on plasma markers of cholesterol absorption and synthesis showed inconsistent findings. In one study, the effect of a long-term aerobic exercise program for 6 months increased plasma campesterol levels, reflecting a higher cholesterol absorption, but did not change plasma markers for cholesterol synthesis in men and women at high risk of developing metabolic syndrome [19]. In contrast, in the current study cholesterol absorption tended to decrease. However, Wilund et al. did not include a no-exercise control group. In the other study with overweight men and women, the effect of alternate day fasting and exercise, composed of combined aerobic and resistance training, was examined [20]. In this randomized controlled trial, no difference in cholesterol absorption markers after 8 weeks intervention between the exercise and control groups was found. However, a significant reduction in cholesterol synthesis in the exercise group was reported, which could also have been due to the alternate day fasting protocol that was followed at the same time [20]. Moreover, only desmosterol levels were decreased, while no changes were observed in lathosterol levels [20]. Desmosterol is an intermediate of the Bloch pathway for cholesterol synthesis and lathosterol of the Kandutsch-Russell pathway [27]. However, this does not necessarily mean that effects of the intervention on cholesterol synthesis were pathway-specific, also because lathosterol is the only validated marker for cholesterol synthesis [9]. In addition, in the recent systematic review, no evidence was found that metabolic aberrations were specifically related to desmosterol and not to lathosterol [17]. Differences in participants characteristics, duration of intervention, methods of dietary control and lack of control group could explain the inconsistency in the effects observed on plasma markers for cholesterol absorption and synthesis. Using stable isotopes, Varady et al. found no effects on cholesterol absorption and synthesis in previously sedentary hypercholesteremic subjects after 6 weeks of endurance exercise [18]. These results agree with the current findings and show that results can be extended to non-hypercholesterolemic sedentary older men using a different approach to estimate fractional cholesterol absorption and synthesis. Despite the 8-week aerobic exercise program, body weight did not change in the exercise period. Dietary habits were comparable between the intervention and control periods, suggesting that any potential loss of fat mass could have been compensated by an increase in fat free mass [28]. Furthermore, factors such as age, sedentary behavior and body composition might affect the exercise intervention, explaining the discrepancy in the current and other studies investigating the effect of exercise on cholesterol absorption and synthesis [29].

Participants can be classified as cholesterol absorbers or cholesterol synthesizers based on their baseline ratios of campesterol to lathosterol [30]. Although not the primary outcome of this study, an exploratory analysis was performed to examine whether the effects of exercise on TC concentrations were different between absorbers and synthesizers. The effect of exercise on TC concentrations did not significantly differ between the subgroups, although we postulated that participants with a higher cholesterol synthesis at baseline (synthesizers) would respond more pronounced to exercise in terms of TC-lowering than those with a higher cholesterol absorption at baseline (absorbers). However, changes in markers for cholesterol metabolism were comparable between cholesterol absorbers and synthesizers and could not explain the slight, non-significant reduction in TC concentrations observed in cholesterol synthesizers, but not in absorbers.

### Study strengths and limitations

A strength of this current study was the inclusion of a sedentary population at a high risk to develop CVD as well as the addition of a no-exercise control period. Furthermore, due to the crossover design, each participant acted as his own control thereby eliminating between-subject variability. There are several limitations in this study. First of all, intake of dietary plant sterols was not known. However, serum plant sterol concentrations were comparable between intervention periods at baseline, suggesting no change in the habitual consumption of plant sterols. Secondly, only male participants were included in the current study but so far there is no indication that non-cholesterol sterols are only valid as markers for cholesterol synthesis or absorption in men and not in women [31]. Lastly, since the current study was not originally designed to investigate the effect of exercise on markers of cholesterol and absorption and synthesis, power calculations were not performed a priori.

## Conclusions

In summary, the 8-week aerobic exercise did not lower serum TC concentrations in older overweight and obese men, but a trend towards a decrease in the cholesterol absorption marker campesterol was found. The cholesterol synthesis marker lathosterol did not change. In future studies, the added value of using non-cholesterol sterol concentrations to classify participants as cholesterol absorbers or synthesizers to increase responsiveness to lifestyle interventions or lipid-lowering therapies needs to be addressed. Nonetheless, increasing physical activity levels is an important approach to reduce CVD risk [32].

## Supplementary Information

**Additional file 1: Supplemental Table 1.** Cholesterol and non-cholesterol sterol concentrations (*n* = 17). **Supplemental Fig. 1.** CONSORT flow diagram of the randomised, controlled crossover study. **Supplemental Fig. 2.** Changes in markers of cholesterol absorption and synthesis during intervention periods for cholesterol absorbers (*n* = 9) and cholesterol synthesizers (*n* = 8) subgroups.

## Data Availability

All data generated or analyzed during this study are included in this article and its supplementary files.

## Abbreviations

CVD: Cardiovascular disease
HDL-C: High-density lipoprotein cholesterol
LDL-C: Low-density lipoprotein cholesterol
TG: Triglyceride
TC: Total cholesterol
IPAQ: International Physical Activity Questionnaire
BMI: Body mass index
Pmax: Maximal power
VO_2_ peak: Peak oxygen consumption
GC-FID: Gas chromatography with a flame ionization detector
