# Supplementary material for: Effects of an 8-week aerobic exercise program on plasma markers for cholesterol absorption and synthesis in older overweight and obese men
Source: Lipids Health Dis. 2021 Sep 21;20:112. doi: 10.1186/s12944-021-01537-2 (PMC8454109; doi:10.1186/s12944-021-01537-2)
Supplement: Supplementary file 1 — Additional file 1: Supplemental Table 1. Cholesterol and non-cholesterol sterol concentrations (n = 17). Supplemental Fig. 1. CONSORT flow diagram of the randomised, controlled crossover study. Supplemental Fig. 2. Changes in markers of cholesterol absorption and synthesis during intervention periods for cholesterol absorbers (n = 9) and cholesterol synthesizers (n = 8) subgroups. [file 12944_2021_1537_MOESM1_ESM.docx]

Effects of an 8-week aerobic exercise program on plasma markers for cholesterol absorption and synthesis in older overweight and obese men

Mashnafi S^1,2^, Plat J^1^, Mensink RP^1^, Joris PJ^1^, Kleinloog JPD^1^, Baumgartner S^1*^

^1^ Department of Nutrition and Movement Sciences, NUTRIM school of Nutrition and Translational Research in Metabolism, Maastricht University, Maastricht, the Netherlands

^2^ Department of Medical Basic Sciences, Faculty of Applied Medical Sciences, AlBaha University, Saudi Arabia

Please address all correspondence to:

*Dr. S. Baumgartner, PO Box 616, 6200 MD, Maastricht, the Netherlands. E-mail: sabine.baumgartner@maastrichtuniversity.nl; Telephone: +31 0 433881305

**Supplemental table 1.** Cholesterol and non-cholesterol sterol concentrations (n=17) at baseline, week 4 and week 8 in control and exercise periods.

|  | **Control** | **Exercise** | **P values** |
| --- | --- | --- | --- |
| Total cholesterol (mmol/l) |  |  |  |
| Baseline | 5.22 ± 1.10 | 5.36 ± 1.03 | 0.102 |
| Week 4 | 5.33 ± 1.29 | 5.42 ± 1.16 |  |
| Week 8 | 5.29 ± 1.09 | 5.31 ± 1.27 |  |
| TC-Campesterol* |  |  |  |
| Baseline | 1.97 ± 0.78 | 2.03 ± 0.74 | 0.494 |
| Week 4 | 2.02 ± 0.78 | 1.90 ± 0.70 |  |
| Week 8 | 2.11 ± 0.82 | 2.00 ± 0.67 |  |
| TC-Sitosterol* |  |  |  |
| Baseline | 1.43 ± 0.59 | 1.46 ± 0.55 | 0.498 |
| Week 4 | 1.47 ± 0.56 | 1.44 ± 0.55 |  |
| Week 8 | 1.48 ± 0.54 | 1.48 ± 0.57 |  |
| TC-Cholestanol* |  |  |  |
| Baseline | 1.39 ± 0.25 | 1.37 ± 0.23 | 0.391 |
| Week 4 | 1.39 ± 0.25 | 1.35 ± 0.23 |  |
| Week 8 | 1.39 ± 0.26 | 1.35 ± 0.23 |  |
| TC-Lathosterol* |  |  |  |
| Baseline | 1.26 ± 0.38 | 1.38 ± 0.42 | 0.120 |
| Week 4 | 1.30 ± 0.41 | 1.41 ± 0.44 |  |
| Week 8 | 1.34 ± 0.39 | 1.36 ± 0.39 |  |

* Expressed as µmol/mmol cholesterol. Data are presented as mean ± SD

**
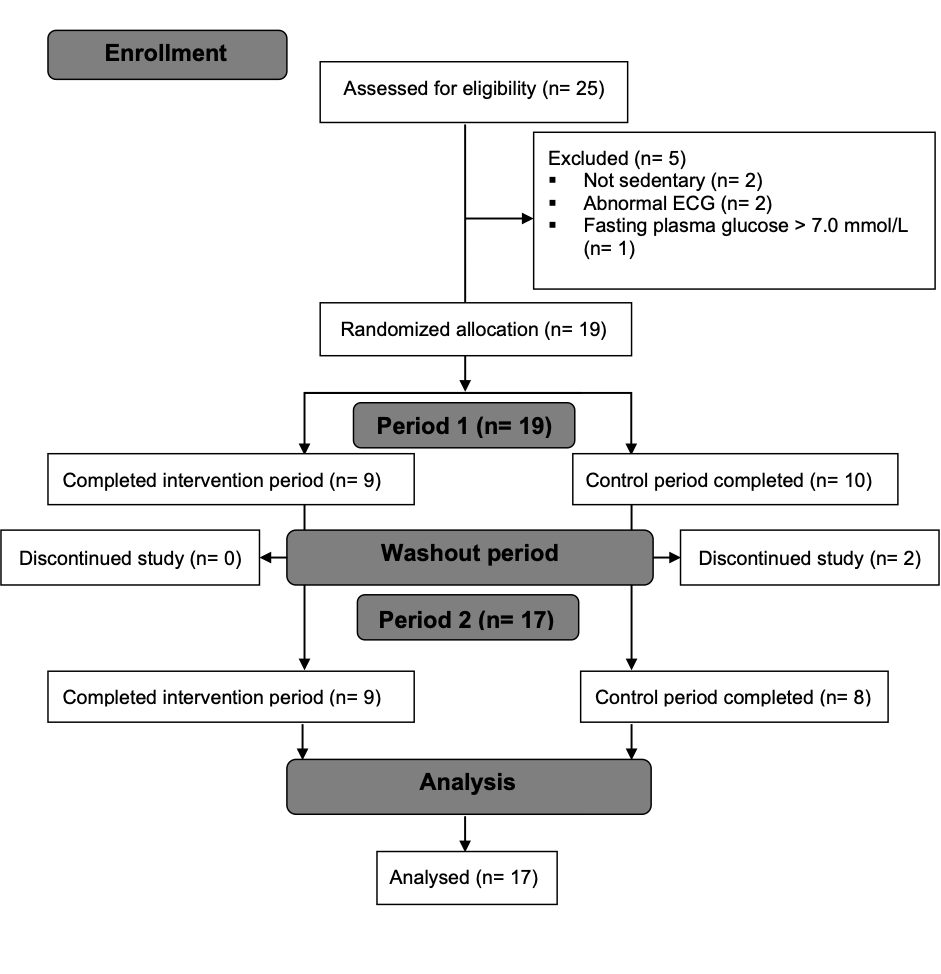
**

**Supplemental figure 1.** CONSORT flow diagram of the randomised, controlled crossover study.

|  |  |
| --- | --- |
|  |  |

**Supplemental figure 2.** Changes in markers of cholesterol absorption and synthesis during intervention periods for cholesterol absorbers (n= 9) and cholesterol synthesizers (n=8) subgroups.

**^A^***P* values for factor effects in cholesterol absorbers.

**^S^***P* values for factor effects in cholesterol synthesizers.
